# Metachronous metastasis- and survival-analysis show prognostic importance of lymphadenectomy for colon carcinomas

**DOI:** 10.1186/1471-230X-12-24

**Published:** 2012-03-23

**Authors:** Tilman Laubert, Jens K Habermann, Claudia Hemmelmann, Markus Kleemann, Elisabeth Oevermann, Ralf Bouchard, Philipp Hildebrand, Thomas Jungbluth, Conny Bürk, Hamed Esnaashari, Erik Schlöricke, Martin Hoffmann, Andreas Ziegler, Hans-Peter Bruch, Uwe J Roblick

**Affiliations:** 1 Department of Surgery, Laboratory for Surgical Research, University of Lübeck, Ratzeburger Allee 160, D-23538 Lübeck, Germany; 2 Institute of Medical Biometry and Statistics, University of Lübeck, Maria-Goeppert-Straße 1, D-23562 Lübeck, Germany

**Keywords:** Colon cancer, Lymph nodes, Metastasis, Prognosis, Survival, Recurrence free survival, Regression analysis

## Abstract

**Background:**

Lymphadenectomy is performed to assess patient prognosis and to prevent metastasizing. Recently, it was questioned whether lymph node metastases were capable of metastasizing and therefore, if lymphadenectomy was still adequate. We evaluated whether the nodal status impacts on the occurrence of distant metastases by analyzing a highly selected cohort of colon cancer patients.

**Methods:**

1,395 patients underwent surgery exclusively for colon cancer at the University of Lübeck between 01/1993 and 12/2008. The following exclusion criteria were applied: synchronous metastasis, R1-resection, prior/synchronous second carcinoma, age < 50 years, positive family history, inflammatory bowel disease, FAP, HNPCC, and follow-up < 5 years. The remaining 421 patients were divided into groups with (TM+, n = 75) or without (TM-, n = 346) the occurrence of metastasis throughout a 5-year follow-up.

**Results:**

Five-year survival rates for TM + and TM- were 21% and 73%, respectively (p < 0.0001). Survival rates differed significantly for N0 vs. N2, grading 2 vs. 3, UICC-I vs. -II and UICC-I vs. -III (p < 0.05). Regression analysis revealed higher age upon diagnosis, increasing N- and increasing T-category to significantly impact on recurrence free survival while increasing N-and T-category were significant parameters for the risk to develop metastases within 5-years after surgery (HR 1.97 and 1.78; p < 0.0001).

**Conclusions:**

Besides a higher T-category, a positive N-stage independently implies a higher probability to develop distant metastases and correlates with poor survival. Our data thus show a prognostic relevance of lymphadenectomy which should therefore be retained until conclusive studies suggest the unimportance of lmyphadenectomy.

## Background

Colon cancer is one of the most common malignant tumor entities in Europe and North America [1]. Patients with synchronous distant metastasis have a significantly worse prognosis than patients diagnosed with localized disease. Despite an increasing motivation to undergo routine screening about 20% of all patients are diagnosed at an advanced tumor stage with manifest metastases (Union Internationale Contre le Cancer (UICC) stage IV). These patients face a 5-year survival rate of around 10% [2,3]. Approximately 40% of patients who initially present without distant tumor growth (UICC stage II and III) will later on suffer from local or distant recurrence [4]. Unfortunately, it still remains clinically impossible to individually predict which patients are more likely to develop distant recurrence after resection of the primary carcinoma.

Colon cancer is staged according to the American Joint Committee of Cancer (AJCC) TNM staging system which reflects tumor invasion, lymph node involvement and existence of distant metastases. Extended lymphadenectomy has long been established as the standard oncologic resection of colon cancer [5]. Hereby, the N-category not only implies the possible indication for an adjuvant treatment in colon cancer but also constitutes an important prognostic factor: different groups have shown a survival benefit for patients with UICC-III and even -II if an extensive dissection and evaluation of harvested lymph nodes were performed [1,6,7]. In contrast, other studies suggest that lymph node dissection might be of minor or no prognostic impact and - potentially - should be omitted: Wong et al. analyzed a cohort of 30,625 colon cancer patients and showed that survival was not significantly different when a median of 6 versus 13 lymph nodes were dissected [8]. Based on an analysis of 16,129 patients Hölzel et al. revealed that the number of positive lymph nodes and occurrence of distant metastases do not correlate [3]. Furthermore, they presented a conclusive statistical model suggesting that lymph node metastases are not capable to set distant metastases. Consequently, they concluded that lymph node resection might be of less clinical importance than currently considered and potentially reflect "overtreatment" [3,9]. One could therefore speculate that similar to the past development of clinical pathways for surgical breast cancer treatment, lymph node resection in colon cancer could soon be performed according to a sentinel concept instead of comprehensive lymphadenectomy as performed today [10].

While many studies do not distinguish between carcinomas of the rectum and the colon, both need to be accounted as different entities regarding anatomic routs of metastasizing, prognosis and therapeutic regimes. In order to determine accurate prognosis and survival rates we therefore considered it necessary to distinguish between both entities and focused exclusively on colon carcinomas in this study. Furthermore, we excluded patients who might bias the analysis due to clinical features known to impact on prognosis: e.g., pre-existing chronic inflammatory bowel disease [11], hereditary colorectal cancer [12,13], and residual tumor tissue after surgical resection [14].

Due to the strict selection of our patient cohort, this study design not only allowed determining the most influencing prognostic factors in a highly selected cohort of colon cancer patients. It was furthermore possible to evaluate the impact of a positive lymph node status on the subsequent development of distant metastases and survival outcome.

## Methods

### Patients

**Figure 1:**
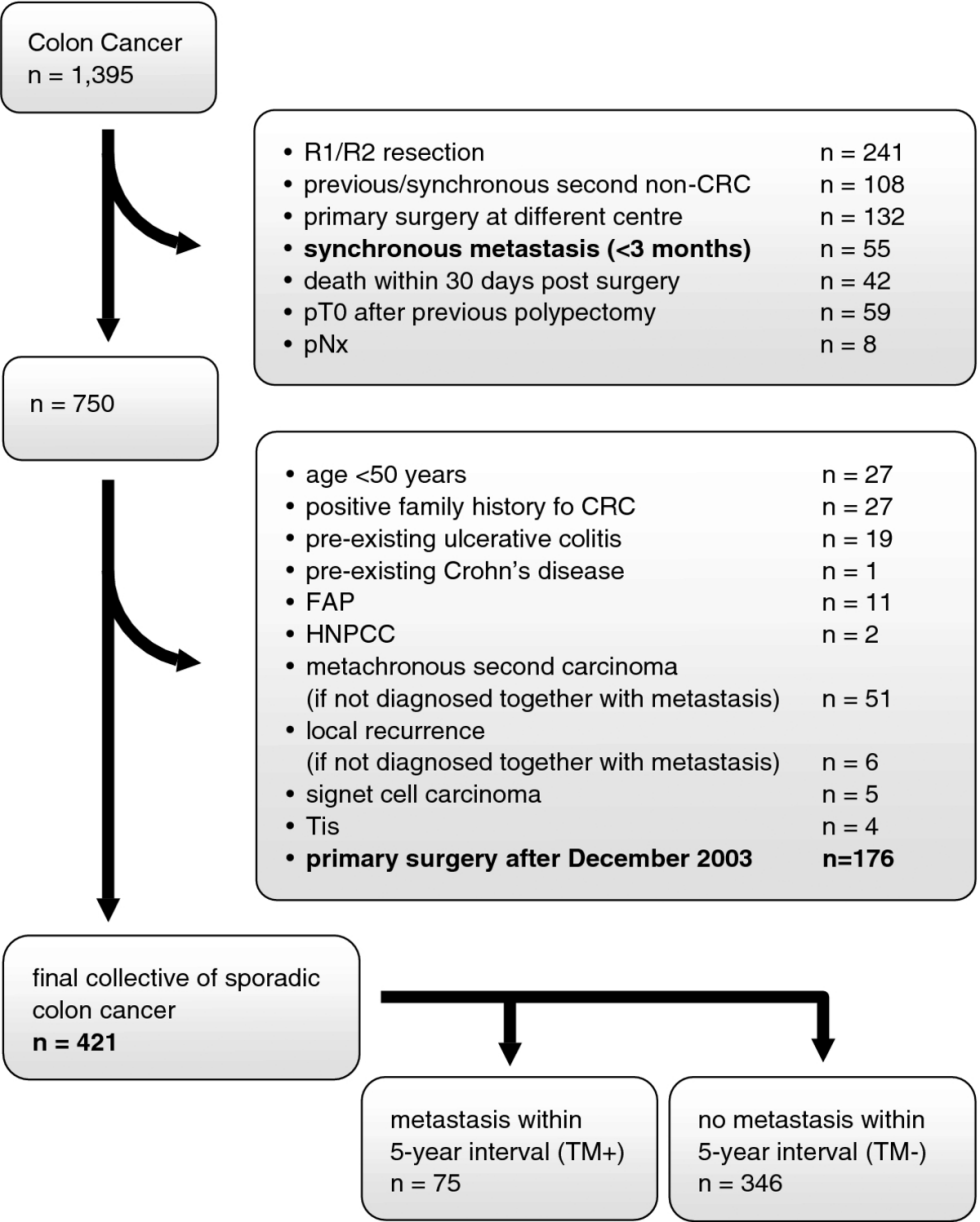
**Exclusion criteria for patients who underwent surgery for Colon Cancer**. The listed exclusion criteria were applied subsequently. CRC: Colorectal cancer, FAP: Familial Adenomatous Polyposis, HNPCC: Heriditary Non-Polyposis Colon Cancer, Tis: Carcinoma in situ.

This study encompassed 1,395 patients who underwent surgery for colon cancer at the Department of Surgery, University Clinic Schleswig-Holstein, Campus Lübeck, Germany, between January 1993 and December 2008. Prospectively documented demographic, clinical and follow-up data were obtained after patients' informed consent and in accordance to the approval of the local Ethical Committee (#07-124). We excluded patients from the cohort of 1,395 patients who had not completed an in-house follow-up of at least 5 years unless an event of either death or diagnosis of metastasis occurred. Thus, median follow-up for the entire collective was 73.6 months (range 1.1-215.5 months). In addition, patients younger than 50 years and patients with synchronous metastases were excluded. "Synchronous metastasis" and "synchronous second carcinoma"were defined as the diagnosis of a distant metastasis or second carcinoma together with or within a three-month interval of the diagnosis of the primary colon cancer. "Metachronous"was defined as an occurrence after a period of three months postoperatively. Additional exclusion criteria known to bias data analysis regarding metastasizing, survival and prognosis of colon cancer were applied (Figure 1). Subsequently, we categorized the remaining patients into those who did develop distant metastasis (TM+, n = 75) or who did not (TM-, n = 346) within a 5-year follow-up. UICC/AJCC stages were defined according to the consensus of 1997 [15].

### Statistics

Continuous variables were expressed as mean ± standard deviation and categorical variables as percent. The Kaplan-Meier curves for TM + vs. TM-, grading 1-3, UICC stage I-IV, N-status 0-2 and right vs. left colon were calculated and assessed for significance by the logrank test. The 5-year survival rates were estimated with the Kaplan-Meier method. The 95% confidence intervals and the p-values were based on an asymptotic approach by using the standard normal distribution. In addition, a Cox regression backwards selection model was used to investigate the effect of clinical and demographic parameters upon recurrence free survival and the occurence of metastasis. For this analysis, initially all variables (sex, age, N-category, T-category, UICC-stage and tumor grading) were taken into account and variables with the highest p-value (for p > 0.1) were excluded subsequently. All results were considered significant with *p *< 0.05. All calculations were performed using SAS software version 9.2.

## Results

### Patient cohorts

We identified 2,570 patients from our colorectal cancer database who underwent surgery between January 1993 and December 2008. Of those, 1,395 were colon cancer patients. The application of exclusion criteria resulted in a collective of 421 patients with 75 presenting with subsequent, metachronous metastasis (TM+) and 346 without (TM-) (Figure 1). Overall, ninety-six patients were staged UICC I, 182 patients UICC II and 143 patients UICC III. For N-stage, there were 278 patients with N0, 92 patients with N1 and 51 patients with N2. For tumor grading, 11 patients presented with G1, 321 patients with G2 and 89 patients with G3. When considering the cohorts of TM + and TM-, both groups presented with comparable clinico-pathological parameters except for differences in T-category, N-category, UICC-stage and age with a higher age in the TM- cohort (Table 1).

**Table 1:** Demographic and clinical data for the cohort which developed metastases within a 5-year interval after primary resection (TM+) and which did not (TM-)

Variable	TM + (n = 75)	TM- (n = 346)	p-value
Age [years (SD)]	68.7 (10.1)	71.5 (10.2)	**0.0222**

Sex			0.4453
female	35 (46.7%)	180 (52.0%)	
male	40 (53.3%)	166 (48.0%)	

Localization			0.7023

right	34 (45.3%)	168 (48.6%)	
left	41 (54.7%)	178 (51.4%)	

T-stage			**< 0.0001**
1	2 (2.7%)	34 (9.8%)	
2	4 (5.3%)	66 (19.1%)	
3	55 (73.3%)	21 (60.1%)	
4	14 (18.7%)	35 (10.0%)	

N-stage			**< 0.0001**
0	29 (38.7%)	249 (72.0%)	
1	24 (32.0%)	68 (19.6%)	
2	22 (29.3%)	29 (8.4%)	

Grading			
1	1 (1.3%)	10 (2.9%)	0.5098
2	55 (73.4%)	266 (76.9%)	
3	19 (25.3%)	70 (20.2%)	

UICC-stage			**< 0.0001**
1	5 (6.7%)	91 (26.3%)	
2	24 (32.0%)	158 (45.7%)	
3	46 (61.3%)	97 (28.0%)	

Continuous variables are expressed as mean (standard deviation) and categorical variables as number (percent). SD: standard deviation. Significant p-values are highlighted in bold

### Survival analysis

**Figure 2:**
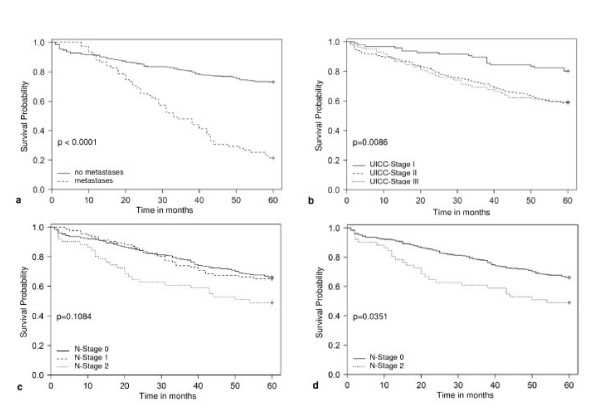
**Kaplan Meier survival curves for the cohorts a) TM- versus TM+, b) UICC-stage I versus II versus III, c) N-stage 0 versus 1 versus 2 and d) N-stage 0 vs. 2**. Significance was determined using logrank-test.

Overall, the 5-year survival rates showed significant differences between UICC stages I and II (p = 0.0001) and between stages I and III (p = 0.0002, Table 2). Kaplan-Meier survival estimation showed significant differences for the three-group comparison of UICC-I, -II and -III (Figure 2b).

**Table 2:** 5-year survival rates (SR) and statistical analyses for comparison of distinct groups of colon cancer patients.

Variable	5-year SR	95% CI	p-value (comparison)	
Metastasis				
yes (TM+)	73%	68-77%	**< 0.0001**	
no (TM-)	21%	13-31%		

Grading				
1	73%	37-90%	0,6578 (1 vs 2)	
2	67%	61-72%		0.1449 (1 vs 3)
3	52%	41-61%	**0.0113 **(2 vs 3)	

N-stage				
0	66%	60-71%	0.7206 (0 vs 1)	
1	64%	53-73%		**0.0230 **(0 vs 2)
2	49%	35-62%	0.0790 (1 vs 2)	

UICC stage				
I	80%	71-87%	**0,0001 **(I vs II)	
II	59%	51-66%		**0.0002 **(I vs III)
III	59%	50-66%	0.9928 (II vs III)	

Localization				
right	59%	52-65%	0.0512	
left	68%	61-74%		

"Metastasis"implies the occurrence of a distant metastasis within a 5-year interval after primary surgery. 5-year SR: 5-year survival rate; 95% CI: 95% confidence interval. α = 0.05. Significant p-values are highlighted in bold

The 5-year survival rates for N-categories were 66% for N0 (95%CI 60-71%), 64% for N1 (95%CI 53-73%) and 49% for N2 (95%CI 35-62%), respectively. A significant difference was found for the comparison between N0 vs. N2 (p = 0.023). In line with these results, Kaplan-Meier survival estimation showed a significant difference exclusively for the comparison of N0 versus N2 (Figure 2c and 2d).

The 5-year survival rates for tumor grading were 73% for grading 1 (95%CI 37-90%), 67% for grading 2 (95%CI, 61-72%) and 52% for grading 3 (95%CI 41-61%), respectively, with a significant difference between grading 2 and 3 (p = 0.0113). The 5-year survival rates for right vs. left colon were 59% (95%CI 52-65%) and 68% (95%CI 61-74%) and did not differ significantly.

When considering the cohorts of TM + and TM-, patients with TM- presented with a significantly higher 5-year survival rate (73%; 95%CI 68-77%) compared to those with TM + (21%; 95%CI 13-31%), as expected (p < 0.0001, Table 2). These results were reflected by Kaplan-Meier survival estimation which showed significant differences for group comparison of TM + versus TM- (Figure 2a).

### Recurrence free survival and metachronous metastases

In order to determine demographic and clinical parameters with significant impact on recurrence free survival we performed a Cox regression backwards analysis taking sex, age, N-category, T-category, UICC-stage and tumor grading into account. This revealed an increasing T-category (HR = 1.84, p < 0.0001), increasing N-category (HR = 1.26, p = 0.033) and male sex (HR = 1.04, p = < 0.0001) as significant parameters with a negative impact on recurrence free survival (Table 3).

**Table 3:** Results of the Cox regression backwards selection analysis for recurrence free survival within a 5-year interval

Variable	Hazard Ratio	95% CI	p-value
sex	1.040	1.024-1.057	**0.033**
N-category	1.261	1.019-1.560	**< 0.0001**
T-stage	1.838	1.422-2.375	**< 0.0001**

Sex, age, N-stage, T-stage, UICC-stage and grading were taken into account. The Hazard Ratio implies a decrease in risk for the female sex and an increase in risk with each additional year of age and for an increase T-stage, given that the other two variables remain constant

Furthermore, we aimed to identify parameters influencing the occurrence of distant metastases. Here, Cox regression backwards selection revealed a significant relevance of an increasing N-category (HR = 1.97, p = 0.0001) and an increasing T-category (HR = 1.78, p = 0.0028) for the occurrence of metachronous distant metastases (Table 4).

**Table 4:** Results of the Cox regression backwards selection analysis for the occurrence of distant metastases within a 5-year interval

Variable	Hazard Ratio	95% CI	p-value
N-stage	1.97	1.49-2.60	**0.0001**
T-stage	1.78	1.22-2.60	**0.0028**

Sex, age, N-stage, T-stage, UICC-stage and grading were taken into account. The Hazard Ratio implies an increase in risk for an increase in N- and T-stage, given that the other variable remains constant

## Discussion

This study evaluates survival and probability for the occurrence of metachronous colon cancer metastasis. For the first time, such an analysis was performed in a highly selected cohort of patients with sporadic colon cancer that passed thorough exclusion criteria. Known clinical parameters were confirmed to be of significant impact for patients' prognosis such as sex, T- and N-category. Furthermore, Cox regression analysis revealed a significant impact of T- and N-category on survival as well as a significant impact of N-category on the risk to develop metachronous metastasis. Only few other studies have performed Cox regression analyses for the same endpoints "survival" and "metachronous metastasis". However, none of these studies was based on a collective with such stringent inclusion criteria [16,17].

Several studies have shown a survival benefit for patients with an extensive examination of lymph nodes for both UICC II and UICC III stage cancers [1,6,7]. The current North American consensus and the German AWMF guidelines demand at least 12 lymph nodes to be retrieved and examined in order to accurately determine the stage of colon cancer [18,19]. However, besides the surgical technique the lymph node yield depends on a variety of variables such as age, immune-response, investigation techniques and tumor location [20]. Despite earlier reports that prognosis improves with increasing lymph node count, the relationship between lymph node yield and survival is not understood and may not be causal but merely statistical in nature. There has been no proof so far that distant metastases can derive from lymph node metastases. The association of tumor-positive lymph nodes with the occurrence of distant metastasis has so far been based on statistical measures and the same accounts for improved survival with an increase of lymph nodes dissected. Still, adequate resection of lymph nodes is also performed in order to control local recurrence as well, especially in stage III colon cancer patients. Studies have shown a survival benefit for patients undergoing complete mesocolic excision which does not imply a particular number of resected lymph nodes but rather aims at the most radical dissection of lymph nodes possibly involved [21,22]. The detection of micro metastases and its association with worse prognosis in terms of occurrence of distant metastases and survival may also not be causal, especially since other studies described contradictory results [23,24]. In fact, several in-vitro and in-vivo studies support the idea that lymph node metastases are themselves not lethal and that surgical lymph node removal should be de-emphasized or omitted [9]. On the other hand, epithelial-mesenchymal transition (EMT) of tumor cells, a process of great importance for the development of distant metastases, is reversed in the metastasis itself (mesenchymal-epithelial transition, MET). After a certain time, cells at the invasive front of the metastasis may repeatedly undergo EMT thus emphasizing their ability to disseminate and metastasize subsequently [25].

Currently, there are no randomized trials or meta-analyses demonstrating a survival benefit of an extensive lymph node dissection in colon cancer. The relevance of radical lymph node dissection has been questioned by other authors [26] and for some tumor entities the extent of radical lymph node dissection was diminished on the basis of clinical studies [27,28]. Based on the colon cancer cohort of the Tumor Registry Munich, Hölzel et al. stated that the risk to develop distant metastases does not correlate with the detection of positive lymph nodes [3]. They also concluded that positive lymph nodes that were not dissected would not contribute to a subsequent occurrence of distant metastases. An important assumption in their conclusions was, however, that a given tumor grows with a constant kinetic. In contrast, our results revealed that besides T-category, particularly N-category is an independent factor with significant impact on the formation of distant metastases.

Many clinical parameters have been described which influence the prognosis of colon cancer. Residual tumor mass (R1 or R2 resection) after removal of the primary malignancy implies a significantly worse prognosis than R0-resected cancers [14]. Inflammatory bowel disease (IBD)-associated colon carcinomas have a worse outcome than colon cancer in patients without IBD [11,29]. Also, hereditary colorectal cancer differs in its prognosis from sporadic colorectal cancer [12,13]. Most studies regarding outcome of colon cancer analyzed large cohorts without exclusion of patients with clinical features known to have an impact on prognosis. Wang et al. excluded patients with prior malignancies, carcinomas located more distally than the recto-sigmoid and those with a histology other than signet cell, mucinous or adenocarcinomas and analyzed a collective of 24,477 CRC patients for lymph node yield and prognosis [30]. They did not exclude patients with potential hereditary malignant disease or IBD. Hashiguchi et al. excluded patients with multiple colon cancers, patients with another synchronous malignancy and patients with a diagnosed polyposis. They analyzed the prognostic significance of the number of lymph nodes examined in colon cancer and included 859 patients from a single centre [31]. In our study we performed all analyses in a thoroughly selected patient cohort thereby minimizing the bias arising from a large but heterogeneous collective. Additionally, in order to address the issue of metachronous distant metastases in relation to the status of lymph nodes dissected during primary surgery we excluded patients who had not completed a 5-year follow-up. Patients who underwent primary resection of colon cancer at a different center were also excluded in order to eliminate possible inter-center variability of patients' prognosis [32].

We found significant differences in survival only for some of the given categories within each parameter such as the difference for N0 versus N2 and grading G2 versus G3. We have to acknowledge the possibility that the lack of significant differences for all categories may be due to the small size of our cohorts. On the other hand, none of the studies showing significant differences for all categories had patients as strictly selected. The cohorts of TM + and TM- differed significantly in T-category, N-category, UICC-stage and age. The difference in age might have biased the results. However, since the TM- cohort was on average older while presenting better survival we argue that the difference is valid and might in fact be larger. The problem of differences in the other variables was addressed by Cox regression analysis. Cox regression analysis showed that T-category and N-category besides sex were parameters with significant impact on 5-year recurrence free survival. Generally, an increase in both N- and T-category is known to be associated with worse prognosis [3]. Only for the regression analysis we used recurrence free survival. For the calculation of survival curves we used overall survival. We have to take into consideration that in this context, analyses of overall survival certainly have shortcomings in comparison to disease specific survival. In order to avoid this dilemma, we limited the survival comparisons to a 5-year interval by censoring all patients with later events. For this period, we estimate the mortality to be primarily related to the pre-existing malignancy.

Our Cox regression analysis revealed that both N- and T-category are parameters with significant impact on the risk to develop distant metastases. Several studies have performed Cox regression analyses with hazard ratios given for each category, i.e. N1 versus N2 and versus N3 [3]. Only a few studies included Cox regression analyses in order to determine which pathological and demographic parameters have the highest impact on the endpoints "death" and "occurrence of a metachronous distant metastasis". Ikeguchi et al. investigated 282 patients with regard to survival and occurrence of metachronous metastases and a possible correlation with various clinic-pathological and cytometric parameters [17]. Lymph node status, tumor location (rectum/colon) and lymphatic invasion were significant factors in logistic regression analysis affecting metachronous hematogenic metastases whereas T-category and ploidy status were not. However, the only exclusion criterion for the collective was adjuvant chemo- or radio-chemotherapy [17]. Heinzerling et al. analyzed 51 distinct clinicopathological parameters addressing the question of metachronous metastasis occurrence [16]. In their multivariate analysis only alcohol intake, patients undergoing abdomino-perineal resection and the use of angiotensin-converting enzyme inhibitor were significantly associated with development of distant disease relapse. In their univariate analysis, however, the dissection of fewer lymph nodes was associated with development of metastasis. The study collective consisted of only 55 patients with carcinoma of both the rectum and the colon and 96% of the patients were males. UICC II stage was the only inclusion criteria and follow-up was three years.

## Conclusion

Much like previous studies we cannot provide any proof for a causal association of lymph node status and a subsequent metastasis. However, our regression analysis shows that lymph node status is of independent prognostic impact for the occurrence of distant metastasis in colon cancer patients. In light of our study, previous clinical studies and molecular investigations it is reasonable to assume that lymph nodes are able to set distant metastases. We therefore suggest that standard dissection of lymph nodes in patients with colon cancer should be continued until further studies suggest otherwise.

## Competing interests

The authors declare that they have no competing interests.

## Authors' contributions

TL, JH, MK: Conception and design of study. TL, JH, EO, RB, PH, TJ, HE: Acquisition of data. CH, AZ, TL, JH, CB, MH, UR: Analysis and interpretation of data. TL, TJ, ES, JH, UR, CB, PH, UR, HB: Drafting and revising of manuscript. All authors: Approval of manuscript version to be published.

## Pre-publication history

The pre-publication history for this paper can be accessed here:

http://www.biomedcentral.com/1471-230X/12/24/prepub
